# Assessment of Monte Carlo algorithm for compliance with RTOG 0915 dosimetric criteria in peripheral lung cancer patients treated with stereotactic body radiotherapy

**DOI:** 10.1120/jacmp.v17i3.6077

**Published:** 2016-05-08

**Authors:** Damodar Pokhrel, Sumit Sood, Rajeev Badkul, Hongyu Jiang, Christopher McClinton, Christopher Lominska, Parvesh Kumar, Fen Wang

**Affiliations:** ^1^ Department of Radiation Oncology The University of Kansas Cancer Center Kansas City KS USA

**Keywords:** Monte Carlo algorithms, lung SBRT, RTOG 0915 compliance criteria, ribs toxicity

## Abstract

The purpose of the study was to evaluate Monte Carlo‐generated dose distributions with the X‐ray Voxel Monte Carlo (XVMC) algorithm in the treatment of peripheral lung cancer patients using stereotactic body radiotherapy (SBRT) with non‐protocol dose‐volume normalization and to assess plan outcomes utilizing RTOG 0915 dosimetric compliance criteria. The Radiation Therapy Oncology Group (RTOG) protocols for non‐small cell lung cancer (NSCLC) currently require radiation dose to be calculated using tissue density heterogeneity corrections. Dosimetric criteria of RTOG 0915 were established based on superposition/convolution or heterogeneities corrected pencil beam (PB‐hete) algorithms for dose calculations. Clinically, more accurate Monte Carlo (MC)‐based algorithms are now routinely used for lung stereotactic body radiotherapy (SBRT) dose calculations. Hence, it is important to determine whether MC calculations in the delivery of lung SBRT can achieve RTOG standards. In this report, we evaluate iPlan generated MC plans for peripheral lung cancer patients treated with SBRT using dose‐volume histogram (DVH) normalization to determine if the RTOG 0915 compliance criteria can be met. This study evaluated 20 Stage I‐II NSCLC patients with peripherally located lung tumors, who underwent MC‐based SBRT with heterogeneity correction using X‐ray Voxel Monte Carlo (XVMC) algorithm (Brainlab iPlan version 4.1.2). Total dose of 50 to 54 Gy in 3 to 5 fractions was delivered to the planning target volume (PTV) with at least 95% of the PTV receiving 100% of the prescription dose (V100%≥95%). The internal target volume (ITV) was delineated on maximum intensity projection (MIP) images of 4D CT scans. The PTV included the ITV plus 5 mm uniform margin applied to the ITV. The PTV ranged from 11.1 to 163.0 cc (mean=46.1±38.7 cc). Organs at risk (OARs) including ribs were delineated on mean intensity projection (MeanIP) images of 4D CT scans. Optimal clinical MC SBRT plans were generated using a combination of 3D noncoplanar conformal arcs and nonopposing static beams for the Novalis‐TX linear accelerator consisting of high‐definition multileaf collimators (HD‐MLCs: 2.5 mm leaf width at isocenter) and 6 MV‐SRS (1000 MU/min) beam. All treatment plans were evaluated using the RTOG 0915 high‐ and intermediate‐dose spillage criteria: conformity index (R100%), ratio of 50% isodose volume to the PTV (R50%), maximum dose 2 cm away from PTV in any direction ($D_{2\text{cm}}$), and percent of normal lung receiving $20\;\text{Gy}\;V_{20}$ or more. Other OAR doses were documented, including the volume of normal lung receiving $5\;\text{Gy}\;V_{5}$ or more, dose to <0.35 cc of spinal cord, and dose to 1000 cc of total normal lung tissue. The dose to <1 cc, <5 cc, <10 cc of ribs, as well as maximum point dose as a function of PTV, prescription dose, and a 3D distance from the tumor isocenter to the proximity of the rib contour were also examined. The biological effective dose (BED) with α/β ratio of 3 Gy for ribs was analyzed. All 20 patients either fully met or were within the minor deviation dosimetric compliance criteria of RTOG 0915 while using DVH normalization. However, only 5 of the 20 patients fully met all the criteria. Ten of 20 patients had minor deviations in R100% (mean=1.25±0.09), 13 in R50% (mean=4.5±0.6), and 11 in $D_{2\text{cm}}$ (mean=61.9±8.5). Lung $V_{20}$, dose to 1000 cc of normal lung, and dose to <0.35 cc of spinal cord were met in accordance with RTOG criteria in 95%, 100%, and 100%, respectively, with exception of one patient who exhibited the largest PTV (163 cc) and experienced a minor deviation in lung $V_{20}$ (mean=4.7±3.4%). The 3D distance from the tumor isocenter to the proximal rib contour strongly correlated with maximum rib dose. The average values of ${\text{BED}}_{3\text{Gy}}$ for maximum point dose and dose to <1 cc of ribs were higher by a factor of 1.5 using XVMC compared to RTOG 0915 guidelines. The preliminary results for our iPlan XVMC dose analyses indicate that the majority (i.e., 75% of patient population) of our patients had minor deviations when compared to the dosimetric guidelines set by RTOG 0915 protocol. When using an exclusively sophisticated XVMC algorithm and DVH normalization, the RTOG 0915 dosimetric compliance criteria such as R100%, R50%, and $D_{2\text{cm}}$ may need to be revised. On average, about 7% for R100%, 13% for R50%, and 14% for $D_{2\text{cm}}$ corrections from the mean values were necessary to pass the RTOG 0915 compliance criteria. Another option includes rescaling of the prescription dose. No further adjustment is necessary for OAR dose tolerances including normal lung $V_{20}$ and total normal lung 1000 cc. Since all the clinical MC plans were generated without compromising the target coverage, rib dose was on the higher side of the protocol guidelines. As expected, larger tumor size and proximity to ribs correlated to higher absolute dose to ribs. These patients will be clinically followed to determine whether delivered MC‐computed dose to PTV and the ribs dose correlate with tumor control and severe chest wall pain and/or rib fractures. In order to establish new specific MC‐based dose parameters, further dosimetric studies with a large cohort of MC lung SBRT patients will need to be conducted.

PACS number(s): 87.55.k

## I. INTRODUCTION

According to the American Cancer Society, approximately 224,390 new cases of lung cancer are expected to be diagnosed in 2016, accounting for about 13.3% of all cancer diagnoses. Lung cancer accounts for more deaths than any other cancer in both men and women in the United States with an estimated 158,080 deaths accounting for about 26.5% of all cancer deaths in 2016.^(1)^ Conventional radiation therapy treatment for early‐stage non‐small cell lung cancer (NSCLC) is considered a viable nonsurgical treatment options for those patients who are medically inoperable because of comorbidities, advanced age, or unwillingness to undergo surgery. However, the five‐year actuarial survival is only 10% to 30%.^2^, ^3^

Stereotactic body radiotherapy (SBRT) with hypofractionated dose schemes using multiple coplanar/noncoplanar beams/arcs and suppression of organ motion has emerged as a promising alternative treatment modality for medically inoperable early‐stage lung cancer patients.^4^, ^5^ SBRT with hypofractionated dose plans significantly reduces the overall treatment time, substantially reduces the treated volume, and improves the local control for these patients.^(4)^ However, treatment planning for lung SBRT patients is challenging due to the involvement of small field sizes and low‐density lung medium (air), which could result in electronic disequilibrium in the regions near low‐density heterogeneity interfaces.^4^, ^5^ Dose calculation algorithms in commercial treatment planning systems (TPS) must have accurate modeling for the tissue density heterogeneity corrections in order to accurately deliver the prescribed dose to patients. At present, the Radiation Therapy Oncology Group (RTOG) lung SBRT protocols require dose planning and monitor units (MUs) calculation for actual treatment plans using dose calculation algorithms that includes tissue density heterogeneity corrections. Dosimetric criteria for RTOG 0915 were established based on the results obtained from non‐Monte Carlo (MC) algorithms such as PB‐hete or superposition/convolutions.^6^, ^7^, ^8^

MC algorithms have been considered as a complex, yet more accurate, method for performing dose calculations in the patient CT datasets. The improved dose calculation is secondary to their ability to accurately simulate radiation transport of a) secondary scatter photons, and b) lateral electron equilibrium. Subsequently, MC algorithms more accurately predict dose distributions within the patient anatomy, specifically, for low‐density tissues such as lung and heterogeneous tissues interfaces. MC‐calculated dose can actually match the physically measured‐dose distributions. Clinically, MC‐based algorithms are now routinely used for lung SBRT dose calculations. Hence, it is important to determine whether MC calculations in lung SBRT meet the RTOG compliance criteria.

There have been several studies reported in the literature on clinical validation of XVMC dose calculations in both homogenous and heterogeneous media utilizing the iPlan RT dose planning system.^9^, ^10^, ^11^, ^12^, ^13^, ^14^, ^15^, ^16^, ^17^, ^18^, ^19^ In recent studies by Sethi and colleagues,^(9)^ XVMC algorithm‐based calculation was scaled using ion chamber and EDR films in various depths in five different phantoms with four different density materials irradiated with 6 MV photons. For heterogeneous lung phantoms, there was excellent agreement (less than 3% difference) between measured and calculated dose profiles with XVMC. Compared to XVMC, PB‐hete calculations overestimated mean PTV measured dose by up to 34%. Most recently, our own clinical experience^14^, ^15^, ^16^ on validating and clinically implementing iPlan XVMC algorithm using Quasar (Modus Medical Devices Inc., London, Canada) phantom study had shown an excellent agreement (within ±2%) between doses calculated using XVMC versus ion chamber measurements for 6 MV‐SRS beam in lung‐equivalent material. In our phantom study,^(14)^ the dose difference between heterogeneities‐corrected PB and measured value was as large as 9% when using Radiological Physics Center (RPC) heterogeneous lung phantom (MD Anderson Cancer Center, Houston, TX). XVMC algorithm not only predicted accurate dose to the isocenter but also at the periphery of tumors where heterogeneities‐corrected PB overestimated the doses at the interfaces, which may be attributed to the lack of electronic equilibrium in the regions near low‐density tissues and heterogeneities interfaces. In our most recent clinical study,^15^, ^16^ using large cohort of lung cancer patients, the mean PTV dose was as high as 15%, on average, when using heterogeneities‐corrected PB algorithm compared to XVMC using the same beam configurations, MLCs and number of MUs. Also, the volume covered by 5 Gy, 10 Gy, and 20 Gy isodose lines of the normal lung were greater than 3.0%, on average, when calculated by PB‐hete compared to XVMC. All the peer‐reviewed literature appears to document the robust experimental validation and clinical implementation of XVMC algorithm, including our own publications.

Recently, many researchers have investigated whether MC‐based dose calculation algorithms can meet the dosimetric criteria set by RTOG 0813 protocol.^20^, ^21^, ^22^, ^23^ For instance, Li et al.^(20)^ evaluated the MC‐based algorithm employed in Monaco TPS (Computerized Medical System, St. Louis, MO) for SBRT lung plans and compared the results against the superposition/convolution algorithm in XiO TPS (Computerized Medical System). A total of 15 NSCLC SBRT patients were used using intensity‐modulated radiation therapy (IMRT) technique. They have presented the dosimetric results from MC‐based algorithm in Monaco that had larger values, 9% (R100%), 12% (R50%), 7% ($D_{2\text{cm}}$), and 18% ($V_{20}$) compared to the ones from superposition/convolution algorithm in XiO TPS. In our most recent clinical study,^22^, ^23^ for centrally located lung SBRT patients, it was observed that iPlan MC dose calculations indicated that the majority (i.e., two‐thirds of the patient population) of our patients had minor deviations in the dosimetric guidelines as established by RTOG 0813 protocol. For 18 centrally located NSCLC patients treated with MC‐based dose calculation, in general, about 6% for R100% and 9% for $D_{2\text{cm}}$ corrections from the mean values needed to be applied to meet the RTOG 0813 compliance criteria in most of those patients. However, no minor deviations were observed for OARs including normal lung, $V_{20}$. To the best of our knowledge, dosimetric evaluation of iPlan MC lung SBRT plan using RTOG 0915 criteria has yet to be reported. One of the requirements of RTOG 0915 protocol is that the plan normalization is to be done to a single point (e. g., an isocenter or a point inside the PTV).^6^, ^7^, ^8^ However, as dose‐volume histogram (DVH) normalization is one of the foremost popular methods used to report the clinical dosimetric parameters, the main purpose of our analysis was to investigate whether those major RTOG 0915 standards could be achieved in patients undergoing lung SBRT with DVH normalization. In this report, the results were presented by applying iPlan XVMC algorithm dose calculation and DVH normalization method for the clinical lung SBRT patients following the RTOG 0915 dosimetric criteria and document ribs dosing.

## II. MATERIALS AND METHODS

### A. Dose calculation algorithm — XVMC

Recently, both heterogeneities corrected pencil beam (PB‐hete) and XVMC algorithms were commissioned and clinically implemented in the Brainlab iPlan RT (version 4.1.2) (Brainlab AG, Feldkirchen, Germany) TPS at our institution. The XVMC was based on the X‐ray Voxel Monte Carlo algorithm^24^, ^25^ that consists of source modeling, beam collimating system modeling, and patient dose computation. The dose calculation parameters for XVMC in iPlan are spatial resolution, mean variance, dose result type, and MLC model. The spatial resolution defines the size of the dose calculation grid, whereas the mean variance estimates the statistical uncertainty of the MC dose calculations. Choosing smaller variance, the dose calculations could be more accurate at the cost of greater computation time. Dose to water and to medium are two options in the dose type. For these clinical lung SBRT patients, we were interested in the average dose within the whole voxel. Therefore, it was default set to dose to the medium. That was the energy absorbed in a small tissue voxel divided by the mass of tissue voxel. Readers are advised to refer to the Brainlab Technical Reference Guide^(26)^ for more details for XVMC algorithm and its clinical implementation.

### B. Patient simulation and target contouring

After obtaining institutional review board (IRB) approval from the University of Kansas Cancer Center, Kansas City, KS, 20 peripherally located Stage I‐II NSCLC patients who underwent MC‐based lung SBRT treatment were included in this retrospective study. The CT simulation was performed on a 16 slice Phillips Brilliance Big Bore CT scanner (Philips Healthcare, Andover, MA) and the patient was immobilized using BlueBAG BodyFIX system (Medical Intelligence, Schwabmuenchen, Germany) in the supine position with abdominal compression. Motion management was done using Philips bellows (Philips Healthcare) for the 4D CT scans. The 4D CT images were acquired with 512×512 pixels at 2 mm slice thickness and 2 mm slice spacing. All 10 phases of DICOM 4D CT datasets were then electronically transferred to the Brainlab iPlan TPS for contouring purposes. Maximum intensity projection (MIP) and mean intensity projection (MeanIP) images were then created in the iPlan TPS and autofused with each phase of 4D CT images. Internal target volume (ITV) was delineated on MIP images of the 4D CT scans. PTV was generated from ITV with 5 mm uniform margin; PTV ranged from 11.1 to 163.0 cc (mean=46.1±38.7 cc). The critical structures, such as bilateral lungs excluding the ITV (total normal lung), heart, ribs, esophagus, and spinal cord, were delineated on the MeanIP images of the 4D CT scans.

### C. Treatment planning and dose calculation

**Figure 1:**
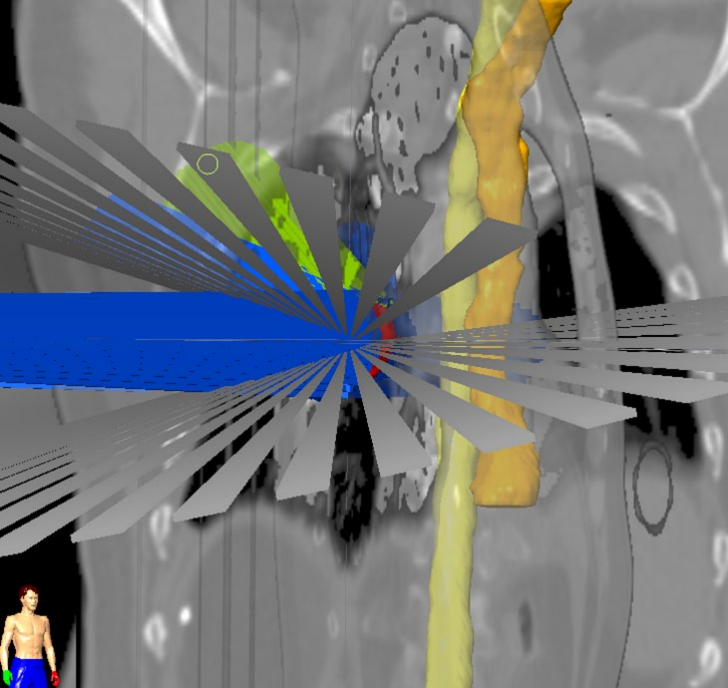
Demonstration of a hybrid arrangement of noncoplanar conformal arcs and static beams for a lung cancer patient treated with SBRT. 3D views of OARs, such as esophagus (dark yellow) and spinal cord (light yellow), are shown with respect to the beam geometry.

Clinically optimal MC SBRT treatment plans were generated using a combination of 3D noncoplanar conformal arcs and nonopposing static beams for the Novalis‐TX linear accelerator (Varian Medical Systems, Palo Alto, CA) with Brainlab system (Brainlab iPlan) consisting of high‐definition multileaf collimators (HD‐MLCs: 2.5 mm leaf width at isocenter) and 6 MV‐SRS (1000MU/min) beam (see Fig. 1). No additional margin for dose buildup was applied at the edges of the MLC blocks beyond the PTV. All treatment plans were calculated on the MeanIP images using XVMC algorithm for heterogeneity corrections with $2.0\times 2.0\times 2.0\;{\text{mm}}^{3}$ dose grid sizes, 2% variance (relative standard deviation of the mean), dose to medium and accuracy optimized for MLC modeling, whereas the MLC is modeled with full tongue‐and‐groove design. It takes into account the air gaps between adjacent leaves. These patients were treated with a dose delivery schema of 54 Gy in 3 fractions (for 1st five patients) and 50 Gy in 5 fractions (for remaining 15 patients) with at least 95% of the PTV receiving 100% of the prescription dose‐DVH normalization. Figure 1 shows the lung SBRT treatment planning setup. Also, for comparison, those plans were recomputed using PB‐hete algorithm using identical beam configurations, MLCs positions, and number of MUs.

In 2, 3, we have shown clinical XVMC‐computed DVHs analyzing the PTV coverage and rib dose for one representative lung SBRT patient. XVMC dose distributions observed in the axial, coronal, and sagittal views for the same patient are demonstrated in Fig. 4. Also, for comparison, DVHs and dose distributions using PB‐hete algorithm are overlaid for the same patient.

Compared to XVMC, PB‐hete algorithm overestimated the mean PTV dose by 15%, on average, and up to 21% in some cases. Therefore, we sought not to compare RTOG dosimetric compliance criteria on PTV coverage, understanding the fact that PB‐hete overpredicted the PTV coverage, underdosing the target volume that was consistent with aforementioned referenced literature^9^, ^10^, ^11^, ^12^, ^13^, ^14^, ^15^, ^16^, ^17^, ^18^, ^19^ comparing the two algorithms.

**Figure 2:**
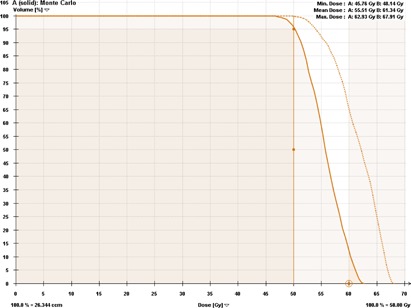
Dose‐volume histogram comparing the PTV coverage predicted by XVMC (solid line) vs. PB‐hete (dashed line). The XVMC PTV coverage showing at least 95% of the PTV is covered by the prescription dose of 50 Gy; R100% was 1.24, mean PTV coverage = 55.5 Gy, and maximum dose = 62.9 Gy. On the other hand, the PB‐hete significantly overestimated the mean PTV coverage by up to 10.5% when compared to XVMC leading to potential underdosing of the tumor volume.

**Figure 3:**
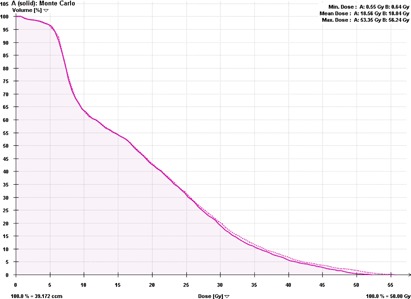
Dose‐volume histogram comparing the dose to ribs predicted by XVMC (solid line) vs. PB‐hete (dashed line) for the same representative patient. For the given same prescription dose, PB‐hete overestimated ribs dose including maximum point dose to ribs by about 3.0 Gy.

**Figure 4:**
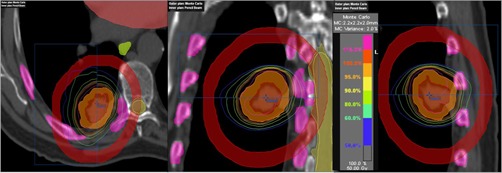
Comparison of the isodose distributions generated via XVMC (outer window) vs. PB‐hete (inner window) on representative axial (a), coronal (b), and sagittal (c) views for a representative NSCLC patient treated with SBRT. Target volumes contoured include ITV (orange, innermost) and followed by PTV (red, outermost). Higher isodose lines, such as 50 Gy (100%), 47.5 Gy (95%), 40 Gy (80%), exhibit sharp dose falloff. The maximum point dose to the PTV was 62.9 Gy (126%) in XVMC vs. 67.9 Gy (136%) in PB‐hete algorithms. The 25 Gy (50%) isodose line was mainly in the ipsilateral lung parenchyma for both algorithms. However, PB‐hete overestimated dose to the PTV as well OARs including normal lung. Other OARs, such as spinal cord, esophagus, heart, and ribs contours, are also shown in the 3D image views. Red color ring was contoured to calculate $D_{2\text{cm}}$ (%).

### D. Treatment plan evaluation

The DVHs of all treatment plans were generated in the Brainlab iPlan TPS and evaluated for the following RTOG 0915 high‐ and intermediate‐dose spillage dose parameters:^(7)^

a) R100%: Ratio of prescription isodose volume to the PTV (conformity index). R100% less than 1.2 is highly desirable; R100%=1.2 to 1.5, acceptable with minor deviations (see Table 1).
b) R50%: Ratio of 50% prescription isodose volume to the PTV. R50% is volume‐dependent (see Table 2).
c) $D_{2\text{cm}}$: Maximal dose 2 cm away from PTV in any direction as a percentage of prescription doses. $D_{2\text{cm}}$ is volume‐dependent (see Table 3).
d) $V_{20}$: Percentage of normal lung receiving dose equals to 20 Gy or more. $V_{20}$ less than 10% is per protocol; $V_{20}$ less than 15% is acceptable with minor deviations.

Furthermore, all the clinical MC plans were evaluated for the relative volume of normal lung receiving 5 Gy dose, dose to <0.35 cc of spinal cord, dose to <5 cc of esophagus, dose to <15 cc of heart, and dose to 1000 cc of total normal lung tissue, as well as rib doses. RTOG dose limits for: a) dose to <0.35 cc of spinal cord >20.8 Gy, b) dose to <5 cc of esophagus >18.8 Gy, c) dose to <15 cc of heart >28 Gy and dose to 1000 cc of total normal lung tissue >12.8 Gy, respectively.

**Table 1:** Evaluation of R100% in SBRT lung plans calculated by iPlan XVMC algorithm. First 5 patients had dose delivery schema of total 54 Gy in 3 fractions, and remaining 15 patients with 50 Gy in 5 fractions.

*Patient No.*	*PTV Vol. (cc)*	*RTOG 0915 Minor Deviation*	*R100% XVMC*
1	11.1	1.2‐1.5	1.19
2	20.1	1.2‐1.5	1.32^a^
3	27.1	1.2‐1.5	1.20
4	50.9	1.2‐1.5	1.13
5	68.1	1.2‐1.5	1.31^a^
6	12.9	1.2‐1.5	1.08
7	15.0	1.2‐1.5	1.34^a^
8	20.1	1.2‐1.5	1.31^a^
9	21.0	1.2‐1.5	1.28^a^
10	26.3	1.2‐1.5	1.20
11	31.2	1.2‐1.5	1.20
12	36.2	1.2‐1.5	1.42^a^
13	37.1	1.2‐1.5	1.32^a^
14	39.7	1.2‐1.5	1.25^a^
15	39.8	1.2‐1.5	1.12
16	53.4	1.2‐1.5	1.30^a^
17	56.5	1.2‐1.5	1.37^a^
18	61.5	1.2‐1.5	1.19
19	132.0	1.2‐1.5	1.20
20	163.0	1.2‐1.5	1.17
AVG	46.1		1.25
STDEV	38.7		0.09

a Data that have minor deviations from RTOG 0915 criteria.

PTV = planning target volume; R100% = ratio of prescription isodose volume to PTV; AVG = average; STDEV = standard deviation.

**Table 2:** Evaluation of R50% in SBRT lung plans calculated by iPlan XVMC algorithm. First 5 patients had dose delivery schema of total 54 Gy in 3 fractions, and remaining 15 patients with 50 Gy in 5 fractions.

*Patient No.*	*PTV Vol. (cc)*	*RTOG 0915 Minor Deviation*	*R50% XVMC*
1	11.1	5.0‐5.9	5.2^a^
2	20.1	4.6‐5.7	4.9^a^
3	27.1	4.5‐5.4	5.2^a^
4	50.9	4.0‐5.0	3.6
5	68.1	3.6‐4.8	4.7^a^
6	12.9	4.7‐5.8	4.2
7	15.0	4.7‐5.7	5.5^a^
8	20.1	4.6‐5.7	5.7^a^
9	21.0	4.5‐5.5	4.4
10	26.3	4.4‐5.4	4.3
11	31.2	4.3‐5.3	4.0
12	36.2	4.3‐5.3	4.7^a^
13	37.1	4.2‐5.2	4.7^a^
14	39.7	4.2‐5.2	4.2
15	39.8	4.2‐5.2	3.7
16	53.4	3.9‐5.0	4.4^a^
17	56.5	3.8‐4.9	4.8^a^
18	61.5	3.7‐4.9	4.0^a^
19	132.0	3.1‐3.9	3.5^a^
20	163.0	2.9‐3.7	3.7^a^
AVG	46.1		4.5
STDEV	38.7		0.6

a Data that have minor deviations from RTOG 0915 criteria.

PTV = planning target volume; R50% = ratio of 50% prescription isodose volume to PTV; AVG = average; STDEV = standard deviation.

**Table 3:** Evaluation of $D_{2\text{cm}}$ in SBRT lung plans calculated by iPlan XVMC algorithm. First 5 patients had dose delivery schema of total 54 Gy in 3 fractions, and remaining 15 patients with 50 Gy in 5 fractions.

*Patient No.*	*PTV Vol. (cc)*	*RTOG 0915 Minor Deviation*	$D_{2\text{cm}}$ *XVMC*
1	11.1	50.0‐58.0	52.2^a^
2	20.1	53.0‐61.9	60.8^a^
3	27.1	55.7‐65.0	57.8^a^
4	50.9	62.2‐77.5	59.4
5	68.1	65.6‐85.1	76.3^a^
6	12.9	50.0‐58.0	49.1
7	15.0	50.9‐59.1	59.0^a^
8	20.1	53.1‐61.9	61.9^a^
9	21.0	53.6‐62.4	52.5
10	26.3	55.3‐64.7	52.7
11	31.2	57.0‐66.8	56.2
12	36.2	58.5‐69.1	69.1^a^
13	37.1	58.8‐69.7	57.5
14	39.7	59.3‐70.8	64.9^a^
15	39.8	59.3‐70.8	53.6
16	53.4	62.6‐78.4	67.4^a^
17	56.5	63.4‐80.2	75.5^a^
18	61.5	64.4‐82.4	64.2
19	132.0	73.7‐91.5	74.9^a^
20	163.0	77.0‐94.0	73.4
AVG	46.1		61.9
STDEV	38.7		8.5

a Data that have minor deviations from RTOG 0915 criteria.

PTV = planning target volume; $D_{2\text{cm}}=\;\text{maximal dose}2\;\text{cm}$ from PTV in any direction as a percentage of prescription dose; AVG = average; STDEV = standard deviation.

### E. Documentation of ribs dose computed by XVMC

The ribs DVH of all MC SBRT treatment plans were also generated and to evaluate the rib dose following RTOG 0915 guidelines.^(7)^ For all plans, doses to <1 cc, <5 cc, and <10 cc of the rib, as well as maximum point doses, were documented as a function of PTV, prescription dose and a 3D distance from the tumor isocenter to the proximity of the rib contour. Also, the average values of ${\text{BED}}_{3\text{Gy}}$ for maximum point dose and dose to <1 cc of ribs computed with XVMC were compared to RTOG 0915 guidelines.

## III. RESULTS

1, 2, 3, 4 present the results of dosimetric parameters from 20 SBRT lung plans corresponding to RTOG 0915 dosimetry evaluation criteria. Only five out of twenty patients met all the RTOG 0915 compliance criteria. Ten of 20 patients had minor deviations in R100%, 13 in R50%, and 11 in $D_{2\text{cm}}$. However, only one patient (#20) associated with the largest PTV had minor violation in normal lung, $V_{20}$.

Ten out of twenty patients had minor deviations in R100%. The mean value of R100% was 1.25±0.09, resulting in a standard deviation (STDEV) of about 7% from the mean value. However, no major violation was observed. Minor deviations in R100% were clinically accepted by the physicians and were treated. The deviations may be primarily due to underlying characteristic behaviors of the XVMC algorithm for dose distribution prediction. The underlying characteristics could be explained by more accurate modeling of a) secondary scatter photons, and b) lateral electron equilibrium, specifically, at the lung and tumor interfaces when using XVMC algorithm.

**Table 4:** Evaluation of normal lung $V_{20}$ in SBRT lung plans calculated by iPlan XVMC algorithm. First 5 patients had dose delivery schema of total 54 Gy in 3 fractions, and remaining 15 patients with 50 Gy in 5 fractions.

*Patient No.*	*PTV Vol. (cc)*	*RTOG 0915 Minor Deviation*	$V_{20}$ (%)^a^ *XVMC*
1	11.1	10‐15	3.6
2	20.1	10‐15	2.2
3	27.1	10‐15	4.2
4	50.9	10‐15	10.0
5	68.1	10‐15	9.9
6	12.9	10‐15	1.5
7	15.0	10‐15	1.8
8	20.1	10‐15	2.1
9	21.0	10‐15	1.6
10	26.3	10‐15	3.1
11	31.2	10‐15	3.3
12	36.2	10‐15	2.3
13	37.1	10‐15	3.3
14	39.7	10‐15	9.2
15	39.8	10‐15	1.1
16	53.4	10‐15	4.7
17	56.5	10‐15	7.4
18	61.5	10‐15	5.7
19	132.0	10‐15	4.3
20	163.0	10‐15	12.8^b^
AVG	46.1		4.7
STDEV	38.7		3.4

a Normal lung $V_{20}$ values ranged from 1.1% to 12.8% (mean=4.7%±3.4%).

b Data that have minor deviations from RTOG 0915 criteria. No minor deviation in normal lung $V_{20}$ from the RTOG 0915 criteria was observed; except for one patient (Patient #20), whose PTV was about 163 cc (the largest PTV recommended by RTOG protocol), had $V_{20}=12.8\%$.

PTV = planning target volume; V20 = percentage of total normal lung minus ITV receiving dose equals larger 20 Gy or more; AVG = average; STDEV = standard deviation.

Thirteen out of twenty patients did not meet the RTOG 0915 R50% compliance criteria, although all of these patients had only minor deviations. The mean value of R50% was 4.5±0.6, resulting in a standard deviation of about 13% from the mean value. Those patients who had minor deviations of RTOG guidelines were deemed clinically acceptable by the physicians and were treated. In those cases, the minor deviations in R50% can be mostly explained by underlying characteristic behaviors of the XVMC algorithm for dose prediction as discussed earlier. They may also be due to the subjective treatment plan review by physicians based on clinical decisions, such as patient geometry and tumor locations that could prevent optimal beam arrangements. For example, recommended partial beam geometry for the peripheral lung tumors or proximity of the ribs from the tumor volume.

Eleven out of twenty patients had minor deviations in RTOG 0915 D2cm compliance criteria. The mean value of $D_{2\text{cm}}$ was 61.9±8.5, resulting in a standard deviation of about 14% from the mean value. However, no major deviations were observed. Those minor deviations could be justified by numerous reasons: a) the underlying characteristics of the XVMC algorithm in predicting more accurate dose distribution in surrounding low‐density lung and heterogeneous tumor interfaces as described above; b) minor deviations in $D_{2\text{cm}}$ were not only associated with large/medium sized tumors but also with a suboptimal conformity index, however, in general, the larger the tumor volume, the harder it is to meet the $D_{2\text{cm}}$ criteria; and c) patient‐specific clinical restrictions preventing optimal beam arrangements during treatment planning and initial subjective treatment plan review by the physician to meet critical structures dose tolerances such as an attempt to reduce the hotspot in ribs.

5, 6 present the results of OARs dose tolerances, such as normal lung $V_{5}$, dose to <0.35 cc of spinal cord, dose to 1000 cc of total normal lung tissue (Table 5), and doses to <1 cc, <5 cc, and <10 cc of ribs as well as maximum point dose to the ribs (Table 6).

The results of our preliminary study show that the RTOG 0915 high‐dose spillage criteria such as R100% was met in 10 out of 20 patients, with 10 minor deviations. The standard deviation of R100% from the mean value was about 7%. However, there was no major deviation in R100% criteria. One of the intermediate‐dose spillage criteria, R50% passed the RTOG guidelines for only seven patients, with 13 minor deviations. The standard deviation of R50% from the mean value was about 13%. Another intermediate dose spillage criterion, $D_{2\text{cm}}$ mostly passed the RTOG guidelines for 50% of the patients only. The mean value of $D_{2\text{cm}}$ was 61.9±8.5 with a standard deviation resulting from the mean value of about 14%. However, for both intermediate‐dose spillage criteria, no major deviations were observed. Only one patient, who had the largest PTV (163 cc), had a minor deviation from RTOG 0915 criteria in normal lung, $V_{20}$. No minor deviations from RTOG 0915 criteria were observed for all other OAR dose tolerances such as dose to <0.35 cc of spinal cord, dose to 1000 cc of normal lung tissue, dose to <15 cc of heart, and dose to <5 cc of esophagus. All these patient plans were clinically accepted by the physicians and the patients were treated.

**Table 5:** Evaluation of normal lung $V_{5}$, dose to 1000 cc of total normal lung tissue and dose to <0.35 cc of spinal cord in SBRT lung plans calculated by iPlan XVMC algorithm. First 5 patients had dose delivery schema of total 54 Gy in 3 fractions, and remaining 15 patients with 50 Gy in 5 fractions.

*Patient No.*	*PTV Vol. (cc)*	*Normal Lung* $V_{5}$ ^a^ *(%)*	*Dose to 1000 cc of Total Lung (Gy)*	*Dose to* <0.35 cc *of Spinal Cord (Gy)*
1	11.1	13.3	1.1	3.0
2	20.1	10.4	1.7	3.5
3	27.1	13.6	2.9	5.6
4	50.9	36.1	2.3	12.6
5	68.1	23.1	2.8	9.0
6	12.9	6.6	1.1	2.6
7	15.0	5.9	0.7	5.2
8	20.1	4.4	0.7	8.1
9	21.0	11.4	1.5	5.4
10	26.3	16.8	2.5	16.4
11	31.2	13.8	1.3	4.0
12	36.2	10.8	2.6	3.9
13	37.1	24.3	4.3	12.8
14	39.7	21.0	1.5	11.7
15	39.8	7.4	6.5	10.4
16	53.4	23.1	3.3	7.6
17	56.5	18.3	1.9	6.1
18	61.5	28.6	3.5	7.9
19	132.0	20.3	3.1	5.5
20	163.0	35.0	3.9	18.3
AVG	46.1	17.2	2.4	8.0
STDEV	38.7	9.2	1.4	4.5

a Normal lung $V_{5}$ values ranged from 4.4% to 35% (mean=17.2%±9.2%).

Note: No minor deviation from RTOG 0915 dosimetric criteria was observed for spinal cord dose and total dose to 1000 cc of normal lung. For all patients, the dose to <5 cc of esophagus and dose to <15 cc of heart were all well below the RTOG requirement, therefore, we did not include those parameters in this table. RTOG minor deviation criteria for dose to <0.35 cc of spinal cord >20.8 Gy, dose to 1000 cc of total normal lung tissue >12.8 Gy, dose to <5 cc of esophagus >18.8 Gy, and dose to <15 cc of heart >28 Gy.

PTV = planning target volume; $V_{5}=\text{percentage of total}$ normal lung minus ITV receiving dose equals to 5 Gy or more; AVG = average; STDEV = standard deviation.

**Table 6:** Documentation of rib doses in MC lung SBRT plans calculated by iPlan XVMC algorithm. First 5 patients had dose delivery schema of total 54 Gy in 3 fractions, and remaining 15 patients with 50 Gy in 5 fractions. For the 1st 5 patients who were treated for a total dose of 54 Gy in 3 fractions, the 3D distance from the tumor isocenter to the proximity of the rib contour was more than 5 cm, except for one patient who had relatively small PTV (20.1 cc, Patient #2), it was 3.5 cm and the associated maximum point dose to ribs was 59.3 Gy.

*Patient No.*	*PTV Vol. (cc)*	*Maximum Rib Dose (Gy)*	*Dose to* <1 cc *of Ribs (Gy)*	*Dose to* <5 cc *of Ribs (Gy)*	*Dose to* <10 cc *of Ribs (Gy)*	*Isocenter to Proximal Rib Contour Distance in 3D (cm)*
1	11.1	41.8	30.2	21.4	12.6	5.6
2	20.1	59.3	42.5	32.2	25.7	3.5
3	27.1	33.1	27.9	25.3	22.2	6.9
4	50.9	45.6	38.7	31.5	27.7	5.4
5	68.1	38.4	33.1	30.5	27.3	7.0
6	12.9	55.5	36.9	10.6	1.1	1.5
7	15.0	54.5	47.9	30.3	20.9	1.7
8	20.1	54.6	44.6	25.7	2.0	1.6
9	21.0	54.4	47.9	28.7	17.4	1.7
10	26.3	53.3	43.5	32.1	24.3	3.9
11	31.2	53.6	42.4	31.4	20.6	3.6
12	36.2	54.1	45.1	32.6	22.9	3.0
13	37.1	51.5	44.7	32.1	23.2	4.4
14	39.7	49.5	42.4	28.1	17.3	5.4
15	39.8	52.4	45.9	33.3	23.3	2.8
16	53.4	54.4	45.8	43.1	28.9	3.6
17	56.5	54.9	44.6	40.6	18.3	3.3
18	61.5	53.3	43.9	36.9	33.6	4.0
19	132.0	52.8	42.4	36.6	19.7	5.0
20	163.0	52.3	42.8	41.6	35.9	5.2
AVG	46.1	50.9	41.6	31.2	21.2	4.0
STDEV	38.7	6.4	5.6	7.3	8.7	1.7

Note: Rib dose values ranged from 35.1 to 59.3 Gy for maximum rib dose (mean=50.9±6.4 Gy) and 27.9 to 47.9 Gy for dose to <1 cc of ribs (mean=41.6±5.6 Gy), respectively; the dose to <10 cc of ribs was less than 36 Gy (Patient #20, who had the largest PTV). The RTOG 0915 suggested doses limit for maximum rib dose <40 Gy, and dose to <1 cc of ribs <32 Gy for a total prescription dose of 48 Gy in 4 fractions.

PTV = planning target volume; AVG = average; STDEV = standard deviation.

## IV. DISCUSSION

Minor deviations from RTOG 0813 compliance criteria while using MC algorithms are very common and were also reported in previous studies.^20^, ^21^, ^22^, ^23^ For example, Li et al.^(20)^ observed that minor deviations from RTOG 0813 compliance criteria (similar to RTOG 0915 dosimetric criteria) on R50%, R100%, and $D_{2\text{cm}}$ using Monaco MC algorithm in XiO TPS. A dosimetric study by Rana et al.^(21)^ using Eclipse RapidArc treatment planning compared the treatment plans computed by analytic anisotropic algorithm (AAA) and Acuros XB for the same number of MUs. The study reported that AAA plans had higher R100%, R50%, and $D_{2\text{cm}}$ when compared to the Acuros XB plans. However, $V_{20}$ of lung was found to be lower in AAA plans. Rana and colleagues also reported minor deviations in R100%, R50%, and $D_{2\text{cm}}$ for both the AAA and Acuros XB plans, with Acuros XB showing minor deviations in fewer cases when compared to AAA. In their study, lower PTV coverage was also reported when using Acuros XB as compared to AAA. In our previous clinical study,^(23)^ for 18 centrally located lung SBRT patients, XVMC dose calculations indicated that the majority (i.e., 67% of patient population) of our patients had minor deviations in the dosimetric guidelines set by RTOG 0813 protocol. Our study suggested that, in general, about 6% for R100% and 9% for $D_{2\text{cm}}$ corrections from the mean value could be applied to pass the RTOG 0813 compliance criteria. One of the differences between our previous study and the Rana study ^(21)^ was the plan normalization method. Dosimetric results from their study were based on the plan normalization at a single point per RTOG requirement, whereas our results were based on DVH normalization. Our findings were consistent with those reported previously.

There have been several studies reported in the literature about chest wall toxicity and rib fracture for lung SBRT patients.^27^, ^28^, ^29^, ^30^ Jain and colleagues^(27)^ compared MC vs. PB algorithms for 20 consecutive patients following RTOG 0915 dosimetric criteria. Their report indicated that MC‐based lung SBRT plans had an increased risk of chest wall and rib toxicity, suggesting that large tumor size and proximity to chest wall predict the highest absolute dose to the rib. From a combined large multi‐institutional study, Dunlap et al.^(28)^ identified the dose‐volume parameters that predict the risk of chest wall pain and rib fracture following lung SBRT. Their results suggested that absolute volume of chest wall receiving greater than or equal to 30 Gy dose correlated with the risk of severe chest wall pain and rib fracture such that the recommended dose was 30 Gy to less than 10 cc of chest wall rib volume. Dosimetric study by Lee et al.^(29)^ described rib fracture and nonrib fracture related pain in large number of lung SBRT patients. By identifying patient, tumor location, dosimetric parameters (i.e., conformity index, gradient, max chest wall dose or dose received by 20 cc of chest wall), and radiologic factors associated with their rib pain or chest wall toxicity, Lee and colleagues concluded that absolute dose of 65 Gy to 20 cc of chest wall volume was associated with radiation‐related chest wall toxicity. Similarly, another dosimetric study presented by Stephans et al.^(30)^ reviewed large cohort of patients and concluded that tumor size and chest wall dosimetry also correlated to late chest wall toxicity. In their study, volume of chest wall receiving 30 Gy through 70 Gy were highly significant for chest wall pain/rib fracture, such that they recommended 30 Gy to 30 cc or less, and/or 60 Gy to 3 cc or less for the chest wall. However, although the literature demonstrates association between rib toxicity and dose‐volume parameters, the majority of studies used non‐MC based methods for rib dose calculation and reporting.

**Figure 5:**
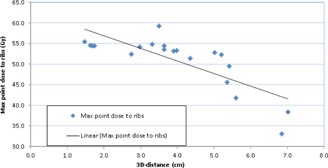
Maximum point dose to ribs computed by XVMC algorithm as a function of 3D distance from the tumor isocenter to the proximal rib contour. For the given prescription dose, the maximum point dose to ribs strongly correlated with 3D distance from the tumor isocenter to the proximal rib contour. The average value of maximum point dose to ribs was 50.9±6.4 Gy (range 33.1‐59.3 Gy).

Our MC‐computed rib doses were on the higher side of the recommended RTOG guidelines, similar to those previously reported.^27^, ^28^, ^29^, ^30^ However, RTOG 0915 report^(7)^ strongly recommended that the lung SBRT treatment plan must be designed not to compromise target dose coverage and not to restrict potential delivery parameters for the sake of meeting the rib dose tolerances. For the given prescription dose, the strong correlation between maximum ribs dose and the 3D distance between to isocenter to the proximity of ribs contour was observed (see Fig. 5). For the maximum point dose to ribs, it was observed that below 2 cm distance it was relatively higher (about 55 Gy); 2 to 5 cm distance had fairly consistent rib doses (from 50 to 55 Gy, except one patient who had PTV to rib contour distance was 3.5 cm and the associated maximum rib dose of 59.3 Gy); and above 5 cm it was easily achievable (i.e., less than 50 Gy).

In general, rib dose should be a function of the 3D distance from the isocenter to the proximity of rib contour, PTV, and prescription dose. However, the larger the target volume, the smaller the overall plan hotspot (therefore, smaller the ribs dose) — suggesting that the larger PTV doesn't necessarily result in higher rib doses regardless of the proximity of the ribs. In general, MC lung SBRT plans are hotter as compared to non‐MC plans. Our previous studies reported that XVMC lung SBRT plans were about 9% hotter, on average, in the heterogeneous phantom^(14)^ compared to XVMC. The PB‐hete algorithm overestimated the mean PTV dose by 15%, on average, and up to 21% in the actual patient plans.^15^, ^16^

**Figure 6:**
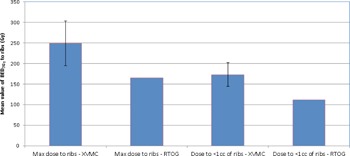
Bar graphs with associated standard deviation error bars comparing the mean ${\text{BED}}_{3\text{Gy}}$ values (n = 20 patients) of maximum point dose and dose to <1 cc of ribs delivered using XVMC vs. RTOG 0915 guidelines. The ${\text{BED}}_{3\text{Gy}}$ for XVMC vs. RTOG 0915 for maximum point dose and dose to <1 cc of ribs were (249±54 Gy vs. 165 Gy) and (173±29 Gy vs. 112 Gy), on average, respectively. Note: Our prescription dose of 54 Gy in 3 fractions (n=5 patients) and 50 Gy in 5 fractions (n=5 patients).

It is apparent from Fig. 6 that delivered ${\text{BED}}_{3\text{Gy}}$ (with α/β=3 Gy) maximum rib dose and dose to <1 cc to ribs was higher than RTOG recommendation. However, this was consistent with RTOG guidelines which stated that lung SBRT plans should be designed not to compromise the PTV coverage, despite proximity of the ribs.

**Figure 7:**
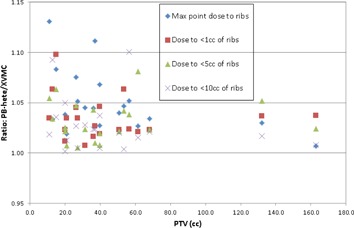
Scatter plot showing ratios of maximum point dose to rib; dose to <1 cc, <5 cc, and <10 cc of ribs calculated by PB‐hete and XVMC algorithms. For the identical beam geometry, MLC margins, and same number of MUs, the PB‐hete algorithm overestimated maximum rib point dose, dose to <1 cc, <5 cc, and <10 cc of ribs by 5%, 3%, 3%, and 3%, on average, respectively, compared to XVMC.

The variation of ratios between PB‐hete vs. XVMC as a function of PTV for maximum point dose, dose to <1 cc, <5 cc, and <10 cc of ribs are shown in Fig. 7. The maximum point dose, dose to <1 cc, <5 cc, and <10 cc of ribs calculated by PB‐hete were uniformly overestimated by 5% (range 1% to 13%), 3% (range 1% to 10%), 3% (range 1% to 8%), and 3% (range 0% to 9%), on average, respectively, compared to XVMC. For smaller tumors and those closer to the chest wall, the magnitude of variations between PB‐hete vs. XVMC computed maximum point dose and dose to <1 cc of ribs were as large as 13% and 10%, respectively. This phenomenon could be explained by the fact that PB‐hete algorithm lacks the ability to account for later electronic equilibrium and secondary scatter photons in dose calculations.

We have reported our MC computed lung SBRT dose parameters to be compliant with RTOG recommendations using dose to the medium dose type, which is considered to be more accurate for lung stereotactic treatment. However, there have been several studies reported in the literature addressing dose specification for radiotherapy treatment,^31^, ^32^ including the AAPM Task Group report.^(33)^ Using Monte Carlo simulation, Ma and Li^(31)^ have reported that conventional photon dose calculation algorithms computed doses were similar to those simulated by Monte Carlo using water with different electron densities that were within 4% differences to doses to medium. However, these doses were significantly larger by up to 11% from doses to water converted from dose to medium following AAPM TG‐105 recommendations.^(33)^ Another earlier study by Siebers et al.^(32)^ used MCNP Monte Carlo code to simulate water‐to‐medium stopping‐power ratio for air, ICRU tissue, lung, soft bone, and cortical bone using ^60^Co to 24 MV beam (see 2, 3). For example, for soft tissues using 6 MV beam, it was reported that the difference between dose to medium and dose to water was approximately 1.0%. However, for soft bone and cortical bone, the dose difference could exceed 3.5% and 10%, respectively. The difference in doses was due to subsequent change of the stopping‐power ratios of tissues compared. If we apply these corrections to convert our MC computed ribs dose, the dose to water could potentially be as high as 10%, in some instances, when compared to dose to medium.

More clinically relevant DVH normalization (i.e., volumetric normalization) techniques have enabled us to further investigate the dosimetric impact of the tumor‐size dependent radiobiological effectiveness of the delivered MC‐based dose to 99% of the PTV (PTV D99) vs. local control for those group of patients.^(34)^ Due to the limitation of CT slice thickness and grid sizes in dose calculation, estimating minimum point dose to the PTV may not reflect clinically meaningful dose coverage, because a tiny voxel stuck between the CT slice could give clinically unacceptable dose to the PTV. Rather we preferred PTV D99 dose coverage for the meaningful estimation of the tumor control probability. While we plan to continue building an MC lung SBRT patient database in our clinic and further investigate RTOG 0915/0813 protocols dosimetric compliance parameters, we would also like to encourage those other institutions that use MC‐based dose calculation algorithms for lung SBRT to do the same. We also plan to investigate and follow up our patients who received MC‐computed dose to the PTV D99 and analyze the local control rates.^(34)^ We will also be clinically evaluate these patients to determine whether delivered MC rib dose correlates with late toxicities, such as severe chest wall pain and/or rib fractures. This data could also potentially include ribs dose reporting methods for MC‐based lung SBRT patients, and could help assess whether to use dose‐to‐medium or dose‐to‐water dose result types based on clinically observed chest wall late toxicity. We recommend multi‐institutional MC lung SBRT studies to assess RTOG guidelines (i.e., met or not met) using varieties of MC‐based dose calculation algorithms. These studies will be critical in establishing new parameters specific for the MC‐based dose calculations in the lung SBRT planning in the future. In addition, the dosimetric impact of various plan normalization techniques, such as point dose vs. DVH normalization per the RTOG 0915 compliance criteria, may need to be further investigated.

## V. CONCLUSIONS

The preliminary dosimetric results for our iPlan XVMC dose calculations with DVH normalization indicates that not all of the RTOG 0915 dosimetric compliance criteria were met in our analyses of the 20 patients undergoing SBRT for lung cancer, although there were no major deviations. However, minor deviations in R100%, R50%, and $D_{2\text{cm}}$ were observed in the majority of the patients (i.e., 75% of the patient population) in one way or the other. When using an exclusively sophisticated XVMC algorithm for dose calculations, the RTOG 0915 dosimetric compliance criteria such as R100%, R50%, and $D_{2\text{cm}}$ may need to be revised. Based on our limited number of patient datasets, in general, R100%, R50%, and $D_{2\text{cm}}$ criteria could be relaxed by about 7%, 13%, and 14%, respectively, to meet the RTOG 0915 criteria. Another less‐desirable option would be to consider rescaling the prescription dose. No further adjustment is necessary for normal lung $V_{20}$, and other OAR dose tolerances such as dose to <0.35 cc of spinal cord and dose to 1000 cc of total normal lung tissue when exclusively using MC‐based dose calculations. All the clinical MC plans were generated without compromising the target coverage; therefore, this resulted in the rib dose being on the higher side of the protocol guidelines. As expected, larger tumor size and proximity to ribs correlated with higher dose to ribs. The average values of ${\text{BED}}_{3\text{Gy}}$ for maximum point dose to ribs and dose to <1 cc of ribs were higher by a factor of 1.5 using XVMC compared to RTOG 0915 guidelines. These patients will be clinically followed to determine whether the delivered PTV dose and the rib dose correlates with local control and late complications, such as severe chest wall pain and/or rib fractures, respectively. In order to establish new specific MC‐based dose parameters, further dosimetric investigations with a larger number of lung cancer patients treated with SBRT are necessary in order to confirm our findings.

## COPYRIGHT

This work is licensed under a Creative Commons Attribution 4.0 International License.
